# Assessing distribution shifts and ecophysiological characteristics of the only Antarctic winged midge under climate change scenarios

**DOI:** 10.1038/s41598-020-65571-3

**Published:** 2020-06-03

**Authors:** Tamara Contador, Melisa Gañan, Gustavo Bizama, Guillermo Fuentes-Jaque, Luis Morales, Javier Rendoll, Felipe Simoes, James Kennedy, Ricardo Rozzi, Peter Convey

**Affiliations:** 1 Sub-Antarctic Biocultural Conservation Program, Universidad de Magallanes, Punta Arenas, Chile; 2 Millennium Nucleus of Invasive Salmonids (INVASAL), Concepción, Chile; 3 Institute of Ecology and Biodiversity (IEB-Chile), Santiago de Chile, Chile; 4 Laboratory for Research in Environmental Sciences (LARES), Faculty of Agricultural Sciences, Department of Environmental Sciences and Natural Renewable Resources, University of Chile, Santiago, Chile; 5 Department of Biological Sciences, University of North Texas, Texas, USA; 6 Department of Philosophy and Religion Studies, University of North Texas, Texas, USA; 7 British Antarctic Survey, Cambridge, UK

**Keywords:** Biogeography, Climate-change ecology, Ecological modelling, Ecophysiology

## Abstract

Parts of Antarctica were amongst the most rapidly changing regions of the planet during the second half of the Twentieth Century. Even so, today, most of Antarctica remains in the grip of continental ice sheets, with only about 0.2% of its overall area being ice-free. The continent’s terrestrial fauna consists only of invertebrates, with just two native species of insects, the chironomid midges *Parochlus steinenii* and *Belgica antarctica*. We integrate ecophysiological information with the development of new high-resolution climatic layers for Antarctica, to better understand how the distribution of *P. steinenii* may respond to change over the next century under different IPCC climate change scenarios. We conclude that the species has the potential to expand its distribution to include parts of the west and east coasts of the Antarctic Peninsula and even coastal ice-free areas in parts of continental Antarctica. We propose *P. steinenii* as an effective native sentinel and indicator species of climate change in the Antarctic.

## Introduction

Antarctica and the sub-Antarctic islands are some of the last wilderness areas remaining on the planet. These remote areas remain, to a great extent, free from direct anthropogenic impacts such as overpopulation and overexploitation of native ecosystems^1^, although they are not immune to wider global anthropogenic processes such as climate change and long-range pollution^2,3^. The high latitude regions of the Antarctic Peninsula, Scotia Arc, and the Magellanic Sub-Antarctic have been amongst the most rapidly warming areas in the world in the second half of the Twentieth Century, showing significant glacier retreat and reduction of snow and ice cover in terrestrial and freshwater ecosystems^3^. While these strong regional warming trends have currently paused, they are predicted to resume through the remainder of the Twenty-first Century^4^. These regions are highly sensitive to environmental change and thus are considered natural laboratories in which to study its effects, at all scales, on their ecosystems and biota^3,5^.

Today, Antarctica remains in the grip of continental ice sheets, with only about 0.2% of its overall area being ice-free^6^, this proportion is somewhat higher in the Antarctic Peninsula region (~3%; British Antarctic Survey unpublished data, Lee *et al*.. 2017). Terrestrial and freshwater ecosystems are generally small and isolated, populated by small invertebrates, lower plants, and microbes^7^. The terrestrial fauna consists only of invertebrates, with just two native species of insects, both chironomid midges (the winged *Parochlus steinenii* Gerke and the brachypterous *Belgica antarctica* Jacobs), and two established invasive species with currently restricted ranges, *Eretmoptera murphyi* (Diptera: Chironomidae) and *Trichocera maculipennis* (Diptera: Trichoceridae)^8^.

Climatic gradients have changed over geological time at different spatio-temporal scales in these high latitude southern regions, shaping the composition and distribution of modern landscapes and their biota^3,9^. The Eocene marked the beginning of the cooling of the Southern Ocean (*ca*. 35 Ma). The gradual breaking of the link between southern South America and the Antarctic Peninsula, around the same time, permitted initiation of circumpolar atmospheric and subsequently oceanic circulation patterns and progressively isolated Antarctic terrestrial habitats from potential sources of colonists from lower latitudes^10^. This, combined with continental cooling, led to the extinction of major groups of organisms, as well as evolutionary radiation amongst survivors (see Convey *et al*. (2018) for an overview of the history of the Antarctic terrestrial biota^11^). However, a rapidly growing body of molecular, phylogenetic, and classical biogeographic evidence strongly indicates that representatives of all extant higher taxonomic groups in Antarctica, including the native chironomid midges mentioned above, survived within the Antarctic continent throughout these environmental changes^12^. The isolation and fragmentation of species’ populations in ice-free areas are amongst some of the evolutionary mechanisms leading to population structuring of contemporary Antarctic taxa^13^. This mosaic of spatial and temporal settings has led to the persistence of a unique biota with varying degrees of tolerance to environmental stresses.

### Global climate change and insects in Antarctica

Climate change is a complex process involving changes in multiple environmental conditions^14^, over a range of timescales, and is not simply an increase in temperature. Thus, the factors limiting a species’ distribution can vary widely over space and time^5,10^. In this context, understanding how multiple environmental factors, both individually and in combination, influence species is essential for predicting how they will be affected over both contemporary and evolutionary timescales.

Ecological Niche Models (ENM) are increasingly used to evaluate the influence of climate change on distribution patterns^15–17^. These models aim to identify areas that are climatically analogous to those within the existing or realised niche of a species. In this context, the integration of life history information and studies on ecophysiology with the results obtained from ENMs provides an effective tool to better estimate and understand the biological consequences of global climate change. Experimental approaches are key to understanding the underlying processes causing biogeographic changes^18^. Therefore, the combination of spatial and physiological data provides an important tool to help identify sentinel species that may provide alarm indicators.

After the middle of the Twentieth Century, the maritime Antarctic experienced significant warming^3^, causing deglaciation^19^ and the appearance of new ice-free areas and freshwater habitats^20^. Maritime Antarctic lakes have experienced extremely rapid physical ecosystem change over the latter decades of the Twentieth Century^21^, even magnifying the very rapid annual air temperature increases over the same period. The lakes, streams and terrestrial habitats that makeup Antarctica’s land-based ecosystems are generally small and isolated, and many of their small invertebrates, lichens, and microbes are found nowhere else on Earth^7^. As Antarctica undergoes some of the most rapid changes worldwide in air temperature, glacial cover, and lake seasonality, these organisms are facing extreme changes in their environments.

Insects are very sensitive to temperature variation, which directly affects their growth rates and ability to survive in a given microhabitat, particularly when temperature variation exceeds their tolerance range^22^. Increasing temperatures influence many elements of insect life histories, from physiology, through development and voltinism, to population dynamics and range^23,24^. The phenology, voltinism patterns, and stress tolerances of insects are critical elements in assessing and predicting the consequences of environmental change in freshwater ecosystems, as well as for their surroundings^25^.

Of the two native Antarctic species of holometabolous insects, the wingless *B. antarctica*, is endemic and widely distributed along the coast of the western Antarctic Peninsula and its offshore islands northwards from the northern tip of Alexander Island to the South Shetland Islands. In contrast the winged *P. steinenii*, while having limited Antarctic occurrence where it is only found on the South Shetland Islands in the maritime Antarctic, is also found on sub-Antarctic South Georgia, and through the Andes of southern South America to 41°S^26^. The larvae and pupae of *P. steinenii* are aquatic, and inhabit permanent lakes in the maritime Antarctic^27^, while the winged adults are terrestrial.

Through this study we aim to integrate new and high-resolution modelling approaches with information on the ecology and physiology of *P. steinenii* to predict shifts in its distribution over the next century. To achieve this objective, we (a) characterize and analyze its present-day distribution across the South Shetland Islands, as well as assessing its ecophysiological characteristics in the laboratory, and (b) create specific climatic variables for the Antarctic at a fine spatial resolution in order to develop ecological niche models for the species, under different climate change scenarios.

## Results

### Characterization of present-day distribution and ecophysiological characteristics of *P. steinenii*

**Figure 1:**
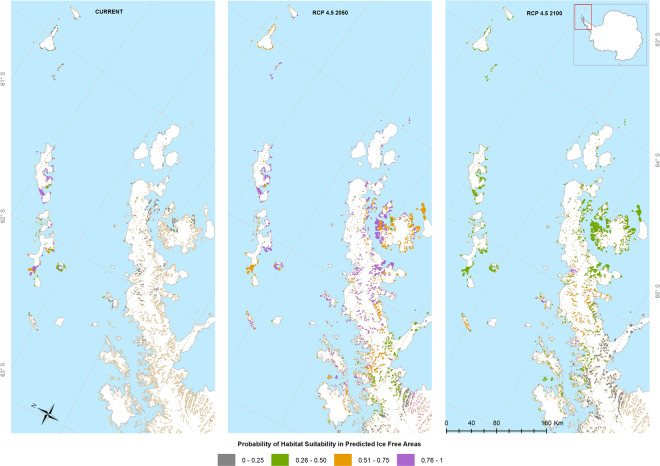
GDLF *Parochlus steinenii* Ecological Niche Model for current and projected distributions for 2050 and 2100 based on RCP 4.5 scenarios, predicted by MaxEnt. This map was created using ArcGIS® software by Esri. ArcGIS® and ArcMap™, ArcGis v.10.1. Base layers: Seamask_high_res_polygon, coastline_high_res_polygon and Rock_outcrop_high_res_polygon available from the Scientific Committee for Antarctic Research (SCAR) Antarctic Digital Database (ADD Version 7; http://www.add.scar.org). These data are licensed according to Creative Commons CC-By – data are free to use, modify and redistribute.

Based on our field observations, we confirmed 58 presence locations for the species in the South Shetland Islands and confirmed that the current distribution of *P. steinenii* is limited to the South Shetland Islands in the maritime Antarctic (Fig. 1). Nonetheless, the MaxEnt procedure identified the existence of further potential contemporary distribution areas in the Trinity Peninsula and James Ross Island, based on suitable climatic conditions. The lowest presence threshold (LPT) obtained was 0.25, and therefore the suitable habitat areas were reclassified into the following four levels: 0–0.25 (unsuitable); 0.26–0.50 (low suitability); 0.51–0.75 (moderate suitability); 0.76–1 (high suitability). The spatial point pattern analysis shows that *P. steinenii* has a significantly clustered distribution (nearest neighbor analysis with wrap-around edge correction, mean distance: 1.9 km, expected distance: 4.7 km, R: 0.40, Z: −8.79, p < 0.0001) throughout the South Shetland Islands (Supplementary Fig. S1). There is a higher density of presence occurrences in King George, Livingston and Deception Islands, which is confirmed by Ripley’s function L(r)-r (Montecarlo Test, p = 0.0093) (Supplementary Fig. S2).

In terms of the Critical Thermal Limits of *P. steinenii*, our assessment of its temperature preferences showed that the average CTmin and CTmax were significantly different between each developmental instar (−2.0 °C, 3.0 °C, and 8.3 °C; and 33.8 °C, 27.5 °C, and 31.4 °C, for larvae, pupae, and adults, respectively) (One-way ANOVA, p < 0.0001, α 0.05) (Table 1, Fig. 4A).

**Table 1:** Summary statistics of CT_Min_ and CT_Max_ of larvae, pupae and adults of *Parochlus steinenii*.

Instar	CT_Min_ (°C)			CT_Max_ (°C)			Thermal range
	Mean ± Std	Lower 95%	Upper 95%	Mean ± Std	Lower 95%	Upper 95%	
Larvae	−2.0 ± 0.8 ^A^	−4.3	0.3	33.8 ± 0.6 ^A^	30.2	32.7	35.8
Pupae	3.0 ± 1.1^B^	0.7	5.3	27.5 ± 0.6^B^	26.6	35.1	24.5
Adult	8.3 ± 1.1 ^C^	6.1	10.6	31.4 ± 0.6 ^C^	26.3	28.7	23.1

ANOVA: F = 21.2, df = 2, *p* < *0.0001*.

Tuckey - Kramer HSD: Adult> pupae> larvae.

ANOVA: F = 27.2, df = 2, *p* < *0.0001*.

Tuckey - Kramer HSD: Larvae> adult> pupae.

^A, B, C^ denote significantly different means (Tukey HSD, P < 0.001, α 0.05).

### Ecological Niche Modelling of *P. steinenii* under different climate change scenarios

The results obtained in the null-models indicate that in all cases the previously generated models were significantly different from chance (see Supplementary Materials for Welch two sample T-test). All models had high AUC, Boyce and TSS values, ranging from 0.78–0.99. We selected the GDLF model (AUC 0.99; Boyce 0.91; TSS 0.93) as the best model to predict the potential distribution of *P. steinenii*, based on the present-day occurrence data, and the ecological and physiological information gathered during this study (Supplementary Table S1). Furthermore, the overlapping GCMs provide a good representation of the extent of each GCM and where they intersect, allowing us to better visualize that GDLF is present in all the possible intersections (see Supplementary Materials, Fig. 4 to 8). The smallest AICc value was β = 1 (see Table S2 in Supplementary Materials). Among the six environmental variables, Temperature Seasonality (Bio4) had the greatest contribution to the distribution model (51.2%) for *P. steinenii*, followed by the Mean Temperature of the Coldest Quarter (36.9%, Bio 11) and the Annual Precipitation (11.5%, Bio12). Together, these three factors explained 96% of the GDLF model distribution.

The Ecological Niche Model (ENM) for *P. steinenii* shows a good match between the species’ present-day Antarctic distribution and the current information generated by the model (Fig. 1). The RCP 4.5 scenario, together with the projection of new ice-free areas (as predicted by Lee *et al*.)^28^, show that for both 2050 and 2100 there are high probabilities of the midge expanding its distribution within the South Shetland Islands and into the northern Antarctic Peninsula (Fig. 1). Specifically, the model predicts that, by 2050, *P. steinenii* will maintain and increase its distribution range in the South Shetland Islands (current distribution area), but it could potentially be found in highly suitable habitats in Antarctic Conservation Biogeographic Region (ACBR) 3 - North-west Antarctic Peninsula^29^. Gibbs, Clarence and Smith Islands show high habitat suitability, as well as the west coast of James Ross and Vega Islands (0.76–1). Additionally, the east coast of Trinity Peninsula, along with Trinity Island, also present high habitat suitability under this scenario.

The model also shows a reduction in probability of suitability in Livingston Island, particularly in Bayer’s Bay (0.51–0.75). At the same time Snow Hill Island, the east coast of James Ross Island and the Nordenskjold coast show moderate habitat suitability. The model also predicts expansion to Elephant Island (north-east of the species’ current distribution) and to Anvers Island (south-west), with moderate habitat suitability. By 2100 the model predicts a decreased probability of habitat suitability within the South Shetland Islands in the species’ current area of distribution, and parts of the Antarctic Peninsula (0.26–0.50). Nonetheless, high or moderate habitat suitability is maintained within Trinity Island and some areas of the west coast of Peninsula (Fig. 1).

**Figure 2:**
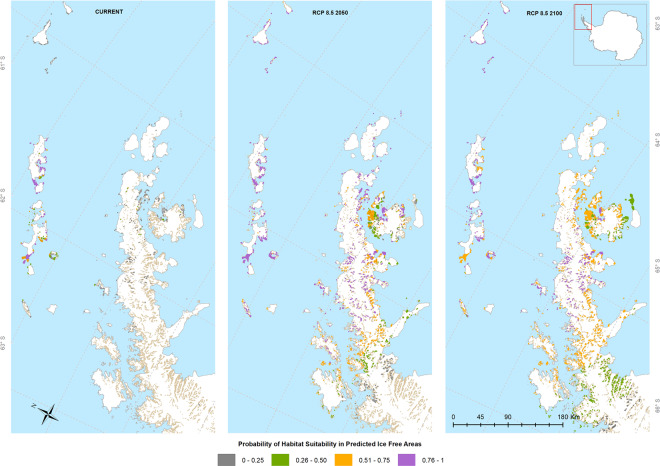
GDLF *Parochlus steinenii* Ecological Niche Model for current and projected distributions for 2050 and 2100 based on RCP 8.5 scenarios, predicted by MaxEnt. This map was created using software ArcGIS® software by Esri. ArcGIS® and ArcMap™, ArcGis v.10.1. Base layers: Seamask_high_res_polygon, coastline_high_res_polygon and Rock_outcrop_high_res_polygon available from the Scientific Committee for Antarctic Research (SCAR) Antarctic Digital Database (ADD Version 7; http://www.add.scar.org). These data are licensed according to Creative Commons CC-By – data are free to use, modify and redistribute.

**Figure 3:**
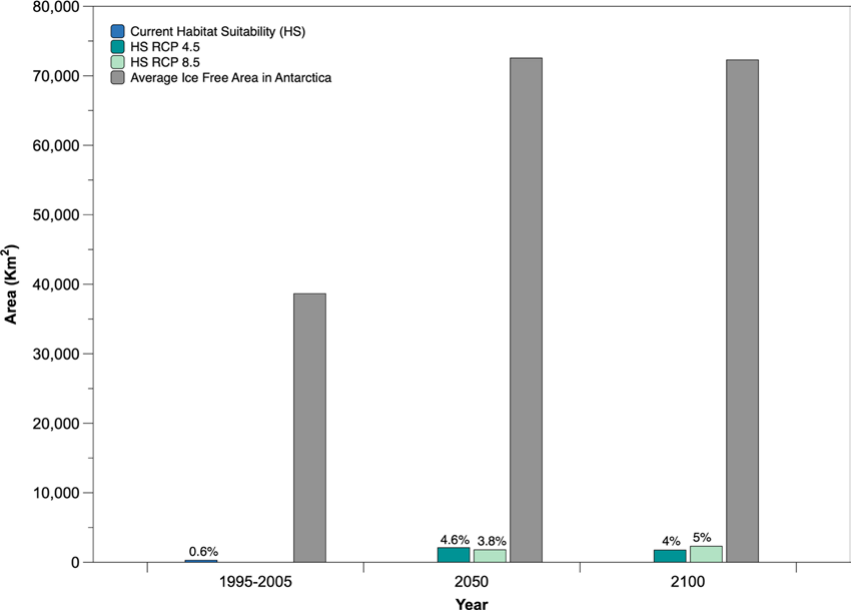
Habitat suitability for *Parochlus steinenii* under IPCC RCP4.5 and 8.5 scenarios in the Antarctic. This graph shows the area suitable for *P. steinenii* in its current distribution (0.6%) in comparison to the area expansion expected under the RCP 4.5 and 8.5 scenarios.

The ENM for RCP 8.5 for 2050 shows a higher persistence of high habitat suitability within the South Shetland Islands, expanding to Smith, Clarence, Elephant and Gibbs Islands (Fig. 2). Highly suitable habitats appear on D’Urville, Dundee, and Bransfield Islands, as well as along the coast of Trinity Peninsula, James Ross Island. However, the probabilities of low suitability habitats existing further south around the Foyn Coast increase (to 0.26–0.50) towards 2100 (Fig. 2). It is also notable that, while the area of potential habitat suitability increases, the degree of suitability is predominantly in the moderate range (0.51–0.75), rather than the highly suitable range (0.76–1). Based on the model approach used here and the extent of ice-free areas, we calculated the total area of potential suitable habitat for this midge. The area of currently suitable habitat is 293 km^2^, which represents 0.6% of the total ice-free area in the Antarctic. The GDLF ENM predicts that the suitable area will increase by 4.6% and 4% in 2050 and 2100, respectively, under RCP 4.5. Under RCP 8.5, the area will increase by 3.8% and 5% for these time periods (Fig. 3).

**Figure 4:**
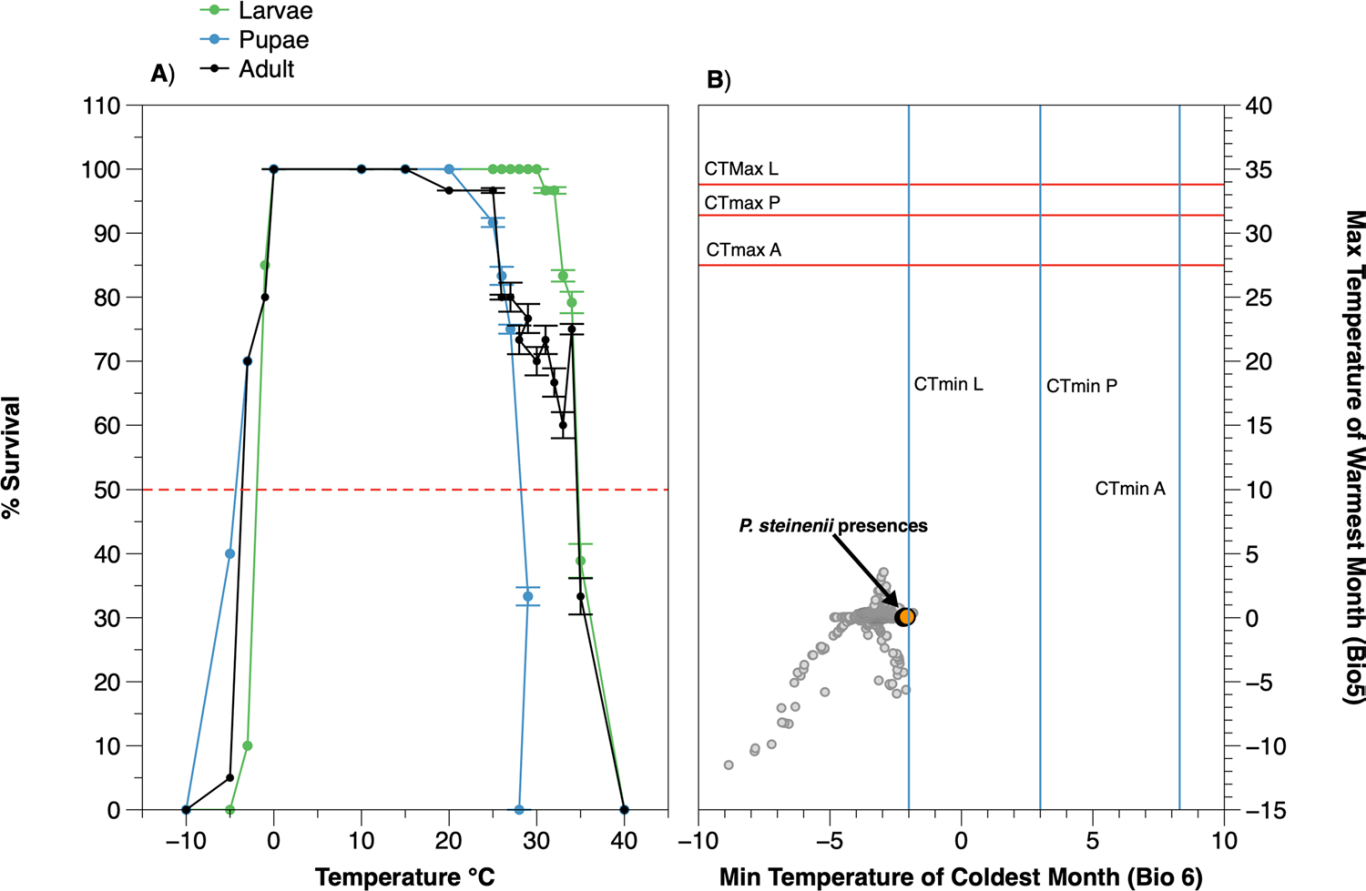
(**A**) Thermal tolerance curves of *Parochlus steinenii* larvae, pupae, and adults. The Critical Thermal Limit at which 50% of the individuals survive is shown by the red-dotted line (CT_50_). (**B**) Niche space of *P. steinenii* within the minimum temperature of the coldest month (Bio5) and the maximum temperature of the warmest month (Bio6) (gray circles). The temperature envelope in which *P. steinenii* is found is shown (orange circles). The red and blue lines represent the physiological Critical Thermal Maximum (CTmax) and Critical Thermal Minimum (CTmin) for the larvae, pupae, and adult developmental stages of *P. steinenii*.

The plot of Minimum Temperature of the Coldest Month (Bio6), portrayed against the Maximum Temperature of Warmest Month (Bio5) (Fig. 4B), shows that the tolerance limits of *P. steinenii* lie towards the minimum critical thermal limit for the larvae. But, in general, the species’ thermal tolerance range for all developmental stages is much wider and exceeds the higher temperature limits of the environmental temperature predicted by MaxEnt (Fig. 4B).

## Methods

### Study area and climate

**Figure 5:**
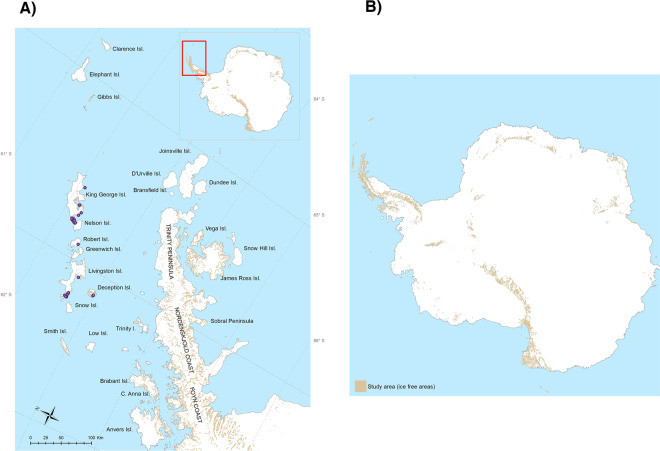
South Shetland Islands in the Maritime Antarctic. Sites assessed with confirmed presences of *Parochlus steinenii* are indicated with purple circles (58 presences in total) (**A**). The total extension of the study area encompasses the ice-free areas of the whole Antarctic continent (**B**). Ice free areas are shown in brown. This map was created using software ArcGIS® software by Esri. ArcGIS® and ArcMap™, ArcGis v.10.1. Base layers: Seamask_high_res_polygon, coastline_high_res_polygon and Rock_outcrop_high_res_polygon available from the Scientific Committee for Antarctic Research (SCAR) Antarctic Digital Database (ADD Version 7; http://www.add.scar.org). These data are licensed according to Creative Commons CC-By – data are free to use, modify and redistribute.

The first stage of this study involved obtaining data across both small-scale microhabitat environmental gradients and larger scale gradients across the South Shetland Islands (63–64°S), the only part of the maritime Antarctic to which *P. steinenii* is native. Fieldwork was conducted during four austral summer seasons (2015/16, 2016/17, 2017/18, 2018/19) during expeditions organized by the Chilean Antarctic Institute (INACH). We surveyed ice-free areas on Deception, Livingston, Greenwich, Robert, Nelson and King George Islands (Fig. 5). These areas are characterized by a geomorphology which includes periglacial landforms, with numerous temporary shallow meltwater ponds and permanent lakes (typically smaller than 100 m^2^), which are ice-covered for 9–10 months each year^30^. The highest elevations reach 167 m (Horatio Stump, Fildes Peninsula) and 266 m (Noel Hill, Barton Peninsula)^30^. Terrestrial habitats in the catchments are characterized by the presence of rich herb-moss and fellfield communities, including the grass *Deschampsia antarctica* and a diverse moss and lichen community^31^. Ecophysiological and life history studies were conducted with populations of *P. steinenii* obtained only on King George Island (Fig. 5).

The climate in the South Shetland Islands is typical of the maritime Antarctic^32^, and is characterized by average summer monthly air temperatures of 0–2 °C during December-March, annual precipitation of *c*. 460 mm and relative humidity of up to 95%^30^. During the Twentieth Century, the strongest warming extended from the southern part of the western Antarctic Peninsula north to the South Shetland Islands in the Peninsula region^33^. The magnitude decreased northwards, away from Faraday/Vernadsky in the Argentine Islands (ca. 65°S)^33^, where the mean annual air temperature rose at a rate of 5.7 ± 2.0 °C per century over this period^34^. The warming trend was not consistent over the annual cycle, with the strongest warming recorded in the winter months, associated with large reductions in winter sea ice extent west of the Antarctic Peninsula, and weaker but still significant trends in the other seasons.

### *Parochlus steinenii* present-day distribution and ecophysiology

**Figure 6:**
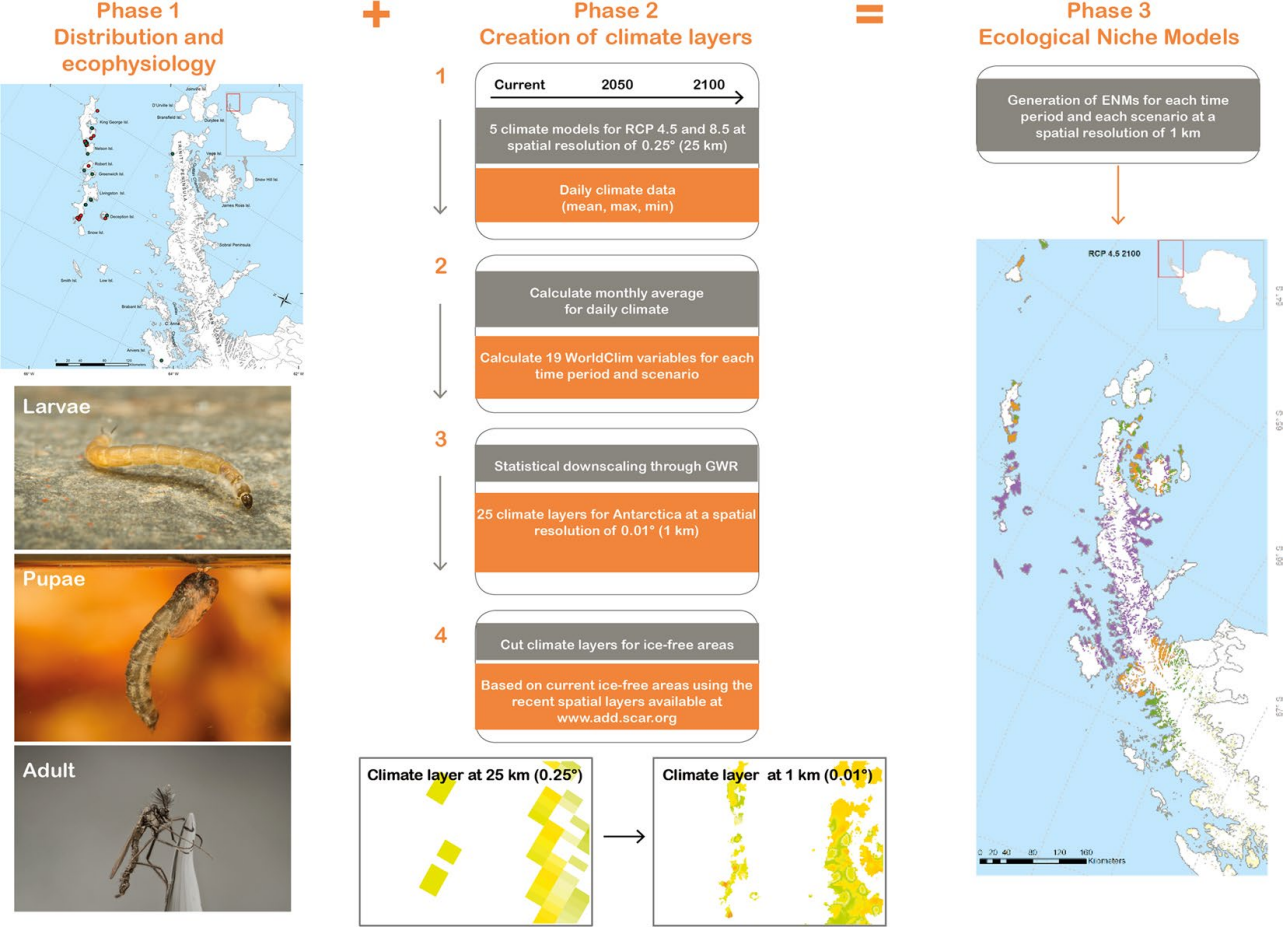
Methodological framework for this study. Phase 1 included assessment of occurrences of *Parochlus steinenii* in its Antarctic distribution, analysis of spatial distribution patterns and thermal ecophysiology of each developmental instar. Phase 2 consisted of the creation of climate layers for the Antarctic, through statistical downscaling, at a spatial resolution of 0.01 (1 km). Phase 3 represents the generation of Ecological Niche Models for each time period and scenario. Photographs of larvae, pupae, and adult Gonzalo Arriagada (CC-BY-4.0). The maps in this figure were created using software ArcGIS® software by Esri. ArcGIS® and ArcMap™, ArcGis v.10.1. Base layers: Seamask_high_res_polygon, coastline_high_res_polygon and Rock_outcrop_high_res_polygon available from the Scientific Committee for Antarctic Research (SCAR) Antarctic Digital Database (ADD Version 7; http://www.add.scar.org). These data are licensed according to Creative Commons CC-By – data are free to use, modify and redistribute.

To characterize the present-day distribution of *P. steinenii* across the South Shetland Islands, we conducted intensive surveys through the ice-free areas accessed (Fig. 6, Phase 1) and sourced all available information from the existing literature^30,35–37^. All accessible sites were searched for a period of 4 to 6 h, defined by climatic conditions and the availability of logistic support. The fieldwork took place during the active season of the adult flies (austral summer), and we also searched for larvae and pupae in water and around the margins of the lakes accessed. We geo-referenced each location examined with a GPSmap 78sc Garmin© unit. To evaluate the thermal environment in which *P. steinenii* develops from egg to adult, we installed temperature data loggers (HOBO® U22 Water Temp Pro V2) in three lakes located on King George Island. These were installed on 8 February 2014 and continue to operate to the present day. These lakes were selected as they host a high abundance of *P. steinenii* and are easily accessible from the Chilean Estación Professor Julio Escudero.

To better understand the present-day distribution, we analyzed the data obtained using spatial point pattern analyses. Spatial point processes are stochastic models that serve as good tools for the analysis of patterns in populations and communities^38^. We conducted a large-scale spatial analysis of the distribution of *P. steinenii* using univariate spatial point process analyses using PAST software^39^. To evaluate the spatial distribution of *P. steinenii*, we used a Complete Spatial Randomness (CSR) model (H_o_: *P. steinenii* has a random spatial distribution in the South Shetland Islands). In this model, spatial points are stochastic and independent, and ‘intensity’ is interpreted as the average density of points per unit area^40^. We used Ripley’s K univariate analysis, with the total area of the islands explored. Results were analyzed using the *L(r) – r* function, which is a transformation of the Poisson *K* function to a straight line, with a constant value = 0, making it easier to assess the deviation from the theoretical function^41^. Monte Carlo tests were conducted (with a 5% probability level) to compare the empirical and the theoretical functions, constructing envelopes under the CSR null hypothesis. The tests reject H_o_ if the observed function lies outside of the critical envelope at any “r” distance value^42^.

#### Ecophysiology: Critical thermal limits

Lower and upper thermal limits of *P. steinenii* were examined using the Critical Thermal Method (CTM), which involves changing temperature at a constant rate until a predefined sub-lethal endpoint (used to estimate lethality) is reached^43^. Larvae, pupae, and adults were collected with an aspirator from Lakes Kitiesh and Langer on King George Island. Live individuals were transported to the laboratory at Estacion Professor Julio Escudero (King George Island) within 2 h of collection. In the laboratory, individuals were acclimated at 8 °C for 24 h in a temperature-controlled cabinet in plastic containers with water, sediment, and small rocks from the collection sites. Larvae (only final instar), pupae and adults were used to conduct experimental assays in the laboratory.

#### Lower thermal tolerance

Six independent experimental assays for each developmental stage were conducted. Each assay contained 10 individual larvae, pupae, or adults. In the case of larvae and pupae, individuals were placed in plastic vials containing water and were submerged in a programmable, recirculating water bath (Lab Companion RW-0252G, Model AAH57003U, Biotech). Adults were placed in plastic containers, each containing a damp filter paper (to avoid desiccation stress). After a 60 min equilibration period at 0 °C, the specimens were cooled to −1 °C at a rate of 0.1 °C/minute. After 1 hour at this temperature, all individuals were removed from the bath and given 24 h to recover at 8 °C in aged tap water, with the exception of adult individuals, which were kept in damp paper towels. After recovery, the target temperature was subsequently lowered by 1 °C and the temperature again reduced at 0.1 °C/min, and the process repeated until the Critical Thermal Endpoints (CTE) was reached (lack of locomotory response to touch with forceps)^44^. The removal procedure was repeated during each trial (24 h recovery) and each individual was assessed for survival and motor function. Those individuals with full motor function were retained for the subsequent trial. The lower sub-lethal temperature was considered to be the temperature after which survival was consistently less than 100% (see Klok and Chown, 2000)^45^.

#### Upper thermal tolerance

Six experimental trials were conducted, each again containing 10 larvae, pupae, or adults. Individuals were gradually warmed at 0.1 °C min^−1^ from a starting temperature of 0 °C. This rate of increase needed to be sufficiently rapid to avoid acclimation, but slow enough to ensure that the core temperature reaction to heating was assessed by observing the behavioral response of the test organisms^43^. Individuals were checked for survival and locomotor function after each increase of 5 °C until reaching 25 °C, after which they were checked at every 1 °C interval. At each temperature checkpoint, observations of each organism were made. When an organism exhibited behavioral indications (lack of movement, lack of response to physical stimulation)^44^ of reaching the critical thermal point, the temperature was recorded, and the organism was removed from the experimental chamber and placed in an aquarium container at 8 °C. Only organisms that recovered from the experimental exposure were included in the subsequent analyses. Successful recovery was defined as the resumption of normal locomotor functions after 24 h recovery time. Differences between the critical thermal limits of each developmental stage (larvae, pupae, and adults), were analyzed using One-Way ANOVAs using PAST software^39^. All experiments were conducted under permits issued by the Scientific Ethics Committee from Universidad de Magallanes and the Bioethics Committee of the Instituto Antártico Chileno (INACH), for INACH project RT_48_16.

### Development of climatic variables for the Antarctic at a fine spatial resolution to develop Ecological Niche Models (ENM) for *Parochlus steinenii*

Following Duffy *et al*.^46^, climatic data were downloaded from the NASA Earth Exchange Global Daily Downscaled Projections (NEX-GDDP; https://cds.nccs.nasa.gov/nex-gddp; dataset (Fig. 6, Phase 2). This array of data contains 21 models and climate scenarios at coarse spatial scale globally (spatial resolution of 0.25°, equivalent to 25 × 25 km). This scale is derived from the General Circulation Model (GCM) that is used under Phase 5 of the Coupled Model Intercomparison Project Phase 5 (CMIP5), which was developed with support of the Fifth Assessment Report of the Intergovernmental Panel on Climate Change (IPCC AR5). These 21 climate models include projections for two scenarios of Representative Concentration Pathways (RCP): RCP 4.5 and RCP 8.5. Each of these projections include daily temperature (minimum and maximum) and precipitation data from 1950–2100. RCP4.5 represents a scenario with a median increase of 2.4 °C set at 650 ppm CO_2_, while RCP8.5 predicts 5 °C above pre-industrial temperatures, and 1370 ppm CO_2,_ by 2100^47^.

In this study, we selected five of the 21 global climate models (ACCESS1.0, BNU-ESM, CESM1-BGC, CSIRO Mk3.6.0 y GFDL-ESM2M), based on those selected by Duffy *et al*.^46^. Because these five models were originally projected in World Geodetic System 1984 (WGS84), we selected and clipped our study area (the Antarctic Continent, the South Shetland Islands) and re-projected the layers to the Antarctic Polar Stereographic (EPSG 3031) Coordinate System Reference (CRS). Then we calculated monthly values for maximum and minimum temperature and precipitation for each of the scenarios (RCP 4.5 and RCP 8.5), obtaining a total of 25 models, as follows: five models for recent time (1996–2005) and 10 models each for 2050 (2046–2050) and 2100 (2096–2100). For each of the 25 models containing mean maximum, minimum and precipitation data, we built 19 analogous climatic variables derived from the WorldClim dataset^48^, using the DISMO package in R^49^.

#### Statistical downscaling from 25 km to 1 km spatial resolution

To increase the spatial resolution of the 19 climatic variables obtained (in each of the 25 models), we conducted a statistical downscaling process (Fig. 6, Phase 2). This process allowed us to increase the spatial resolution from 25 to 1 km, a more appropriate and accurate scale for the evaluation of impacts of climate change on natural habitats and biota^50^. Downscaling was achieved using a multivariable Geographically Weighted Regression (GWR) downscaling method. This type of method is useful in the development of spatio-temporal regressions affected by the phenomenon of parametric instability, providing suitable results to allow the generation of maps with the variables and parameters adjusted to different scales^51^. Other methods use simple regressions to better understand the behavior of spatial variables; nonetheless, the coefficients for such equations do not vary spatially. In this study, we used the Digital Elevation Model (DEM) for the Antarctic at 1 km resolution, which was obtained from the Combined ERS-1 Radar and ICESat laser Satellite Altimetry (available from NISIDC’s FTP site: ftp://sidads.colorado.edu/pub/DATASETS/DEM/nsidc0422_antarctic_1km_dem/). The spatial coefficients obtained through the GWR were interpolated using the Inverse Distance Weighted (IDW) interpolation to the 4th power in order to apply them to the DEM predictor. All statistical analyses were conducted using the R packages Hexbin, hydroGOF, Topmodel and GWmodel. For more details on the downscaling methodology and the GWR, see Fotheringham *et al*.^51^, and Morales *et al*.^52^.

From the distribution data obtained in the field, we obtained a total of 58 confirmed occurrence sites for *P. steinenii* (Fig. 6, Phase 3). Climate suitability for *P. steinenii* was modelled under current and future climates through the application of ENMs. To provide a limit to the true distribution of *P. steinenii*, the spatial background used for this study was the current ice-free areas^28^ (Fig. 5A,B), using the recent spatial layers available in the Scientific Committee for Antarctic Research (SCAR) Antarctic Digital Database (ADD Version 7; http://www.add.scar.org). To avoid co-linearity between the 19 WorldClim variables obtained, we ran a correlation test using ENMTools software^53^, avoiding the incorporation of pairs of colinear bioclimatic variables (Pearson’s r ≥ 0.7). Using this procedure, the following variables were selected: (1) Annual Mean Temperature (Bio1), (2) Temperature Seasonality (standard deviation *100) (Bio4), (3) Max Temperature of Warmest Month (Bio5), (4) Min Temperature of Coldest Month (Bio6), (5) Mean Temperature of Coldest Quarter (Bio11), and (6) Annual Precipitation (Bio12). The ENMs for *P. steinenii* were calculated in the current period and projected to future scenarios (2050 and 2100 for RCP 4.5 and RCP 8.5). Each model was adjusted using maximum entropy algorithms in MaxEnt 3.33e software^54^. The MaxEnt software has been frequently used to simulate shifts in species ranges under current and future climate scenarios^55^. It is based on a probabilistic framework, assuming that the incomplete empirical probability distribution (based on species presences), can be approximated by a probability distribution of maximum entropy, which represents a species’ potential geographic distribution^56^. The MaxEnt approach has a better performance for datasets based on a limited number of occurrences^57,58^, with a combination of high spatio-temporal predictions^59^. The regularization multiplier used for modelling was β = 1, this was decided after comparing the models obtained with different β values (0.25; 0.5; 0.75; 1.0; 1.25; 1.5; 1.75; 2.0). To achieve this, we used the corrected Akaike information Criterion (AICc) available in the software ENMTOOLS version 1.4.4^53^; and the smallest AICc value was considered^60^. We used 75% of the available data as training data, and the remaining 25% were used to evaluate the model with 50 replicates and 10,000 pseudo-absences. To quantify the predictive performance of presence-only models, we performed null-models to assess if the models developed differ significantly from those that would be expected by chance. To achieve this, we used the methodology proposed by Raes and ter Steege^61^. To choose the most adequate model we used the indices AUC^62^, True Skills Statistics (TSS)^63^ and Boyce^17,64^. We also used the confirmed contemporary distribution and ecophysiological data obtained in order to select the best ENM for *P. steinenii* in its Antarctic distribution. TSS and Boyce values can range from −1 to +1 where the value of +1 indicates perfect model performance, a value ~ 0 is not better than random^63^, and negative values indicate reverse models. AUC values range from 0 to 1, and a value of 0.5 or below indicates that the model is not better than random^17,64^. Furthermore, we overlapped the GCMs to better visualize the intersections between the models and provide a better view on the effects of the different GCMs. The smallest AICc value was β = 1 (see Table 2 in Supplementary Materials). MaxEnt also computes response curves showing how the predictions depend on the variables, which greatly facilitates the interpretation of a species’ ecological niche and its defining or limiting environmental factors^56^. To assess how the prediction relates to the ecophysiology of the species under the different climate scenarios, we plotted Bio5 and Bio6 with the Critical Thermal Limits obtained for *P. steinenii*. Finally, we assigned habitat suitability levels by choosing the “lowest presence threshold” (LPT). The LPT is conservative, as it identifies a) pixels predicted as being at least as suitable as those where a species’ presence has been recorded, and b) the minimum predicted area possible whilst maintaining zero omission error in the training data set^65^. From the LPT, four suitability habitat probability levels were derived: unsuitable, low suitability, moderate suitability, high suitability. Finally, we created maps of the ENMs using ArcMap 10.1 and QGIS v2.18 “Las Palmas” (QGIS Development Team 2016. QGIS Geographic Information System. Open Source Geospatial Foundation Project. http://qgis.osgeo.org).

## Discussion

In this study, we aimed to better understand how the native winged midge, *Parochlus steinenii*, may respond to climate change in Antarctica, by combining and integrating physiological and ecological information along with an extensive compilation of the species’ current distribution in its native Antarctic range.

First, we identified that the distribution of *P. steinenii* was better explained by temperature seasonality (Bio4) and the mean temperature of the coldest quarter (Bio11). Insects are generally sensitive to thermal variation. Accumulating evidence suggests that metabolism-linked changes in aquatic insect phenology may affect the synchronization between life history stages and the availability of food or habitat, leading to temporal de-coupling that in turn could result in population crashes or extinctions^23^. Bradie and Leung^66^ showed that, from 400 distinct environmental variables in 2040 ENMs, the most important variables explaining the distribution of the class Insecta were temperature, precipitation, and habitat patchiness.

The ENM obtained for the current distribution of *P. steinenii* is limited to ACBR 3, and represents well the species’ present day Antarctic distribution, as confirmed by our field campaigns and the available literature^29,35,67^. The prediction also includes a small proportion of currently unoccupied suitable habitat in the northern Antarctic Peninsula, particularly on James Ross Island. Nonetheless, there is no evidence from paleolimnological studies of *P. steinenii* occurring in this area, at least since LGM ice retreat^68,69^. From our projections, we can conclude that the area of suitable habitat for *P. steinenii* will increase by 4.6% and 4% (for scenarios RCP4.5 and 8.5 by 2050, respectively, including expansion into ACBR 1. By 2100, our models predict that suitable habitat will increase by 3.8% and 5%, respectively, with a potential expansion in distribution to include ACBRs1, 2 and 3.

How might *P. steinenii* colonize these new suitable habitats in the Antarctic Peninsula and elsewhere in the continent, especially given that paleolimnological evidence suggests that the species has not previously occurred in these areas^68,70^? Dispersal is a major life history trait^71^ and is particularly important in changing and extreme environments, and in areas where landscapes are becoming increasingly fragmented, where movement between local populations plays a vital role in the persistence and the dynamics of entire meta-populations^71^. In this context, our study considers a theoretical full-dispersal scenario. In this, dispersal across the Antarctic could be facilitated by either natural or human-induced actions, with species from adjacent areas crossing biogeographic boundaries and establishing in new ecosystems. When long distances have to be covered, as is the case with movement into Antarctica from lower latitudes, and even from the sub-Antarctic islands, such movements are today generally mediated by human activity^72^. *P. steinenii* has a patchy and fragmented distribution and, although it is a flying insect, its dispersal across larger regions may be limited by abiotic factors such as the strong winds and cold temperatures of the Antarctic. In this context, natural dispersal from the South Shetland Islands to other regions may be limited, unless it is facilitated by other vectors such as birds (e.g. skuas) or human transport. The latter in particular may inadvertently move organisms far beyond their natural dispersal ranges^73^. Human activity (i.e. movement by cargo, ship, aircraft and overland travel) in the Antarctic has a substantial potential for transporting species from one biogeographic region to another. Thus, although *P. steinenii* has not been historically found on the mainland of Antarctica, it could potentially expand its distribution to ACBRs located in the Antarctic Peninsula (ACBRs 1, 2, and 3). This becomes particularly plausible for these ACBRs, as they include some of the areas with the highest indices of human footprint and activity/connectivity between each other^73,74^.

If such transfer does occur, by whatever means, will *P. steinenii* be able to persist in these new regions? Our data on the species’ thermal physiology and habitat preferences, along with the high reproductive output reported by Hahn and Reinhardt^30^, and the lack of native potentially competing or predatory species in the new regions, suggest that this species could rapidly colonize habitats that become available. In general, climatic regimes influence species distributions, often through species-specific physiological thresholds of temperature and precipitation tolerance^75^. Here, consistent with the results reported by Shimada *et al*.^76^ we found that *P. steinenii* is intolerant to freezing and is currently living near its coldest temperature limit, but otherwise has a wide thermal tolerance range in all of its life stages (Fig. 4B). It is found in terrestrial and aquatic environments, depending on life stage. The larvae and pupae are aquatic and inhabit deeper permanent lakes, while adults are terrestrial and are found in very high abundances and density (~600–800 individuals cm^−2^) during the Antarctic summer at the edge of lakes and streams^27,77^, where copulation and oviposition occur. According to our field observations and to Hahn and Reinhardt^30^, *P. steinenii* is likely to mate multiple times. This would suggest that there is a high probability that any dispersing adult females have an adequate sperm supply to found new populations in new suitable habitats. Furthermore, the recent increase in air temperatures in the Maritime Antarctic, especially during winter^34^, combined with increasing precipitation^3^, will possibly alter the duration and thickness of ice cover on freshwater lakes, as well as water level variability^21^. If the predictions generated by recent climate models are correct, freshwater ecosystems on the Antarctic Peninsula may be harshly affected^21,78^, thus affecting the persistence of *P. steinenii* in its current distribution. In this context, *P. steinenii* can be taken as an effective sentinel of climate change in Antarctic terrestrial and aquatic ecosystems, as fluctuations in the thermal environment may significantly impact its current distribution, leading to important ecosystem changes in the Antarctic regions in which it is found.

## Supplementary information

Supplementary Materials.

## Data Availability

The climate layers generated during the current study are available from the corresponding authors on request. All occurrence data for *Parochlus steinenii* will also be able available at GBIF (www.gbif.org).
