# Supplementary material for: Assessing distribution shifts and ecophysiological characteristics of the only Antarctic winged midge under climate change scenarios
Source: Sci Rep. 2020 Jun 3;10:9087. doi: 10.1038/s41598-020-65571-3 (PMC7270094; doi:10.1038/s41598-020-65571-3)
Supplement: Supplementary file 1 — Supplementary Materials. [file 41598_2020_65571_MOESM1_ESM.docx]

**Assessing distribution shifts and ecophysiological characteristics of the only Antarctic winged midge under climate change scenarios**

Tamara Contador^1,2,3*+^, Melisa Gañan^*1+^, Gustavo Bizama^4^, Guillermo Fuentes-Jaque^4^, Luis Morales^4^, Javier Rendoll^1,3^, Felipe Simoes^7^, James Kennedy^1,5^, Ricardo Rozzi^1,3,6^, Peter Convey^7^

^1^Sub-Antarctic Biocultural Conservation Program, Universidad de Magallanes, Punta Arenas, Chile

^2^Millennium Nucleus of Invasive Salmonids, INVASAL, Chile

^3^Institute of Ecology and Biodiversity (www.ieb-chile.cl), Santiago de Chile, Chile

^4^Laboratory for Research in Environmental Sciences (LARES), Faculty of Agricultural Sciences, Department of Environmental Sciences and Natural Renewable Resources, University of Chile, Santiago, Chile

^5^Department of Biological Sciences, University of North Texas, Texas, USA

^6^Department of Philosophy and Religion Studies, University of North Texas, Texas, USA

^7^British Antarctic Survey, Cambridge, UK

*Corresponding author: [tamara.contador@umag.cl](mailto:tamara.contador@umag.cl), melysa_gm@yahoo.es

^+^ These authors contributed equally in this work

Supplementary material

Table 1. Models generated and performance indices obtained for the potential distribution of *Parochlus steinenii* in the maritime Antarctic. The selected model (GDLF) for the potential distribution of the midge is shown in bold.

| Model | Boyce | AUC | TSS | Average |
| --- | --- | --- | --- | --- |
| ACCESS1 | 0.86 | 0.99 | 0.83 | 0.90 |
| BNU | 0.81 | 0.99 | 0.88 | 0.90 |
| CESMI | 0.95 | 0.99 | 0.90 | 0.95 |
| CSIRO | 0.78 | 0.99 | 0.90 | 0.89 |
| GDLF | **0.91** | **0.99** | **0.93** | **0.94** |

Table 2. Values of Corrected Akaike Information Criterion (AICc).

| β value | AICc score |
| --- | --- |
| 0.25 | 628.841 |
| 0.5 | 618.088 |
| 0.75 | 594.426 |
| 1.0 | 588.236 |
| 1.25 | 596.861 |
| 1.5 | 602.563 |
| 1.75 | 602.886 |
| 2.0 | 608.490 |

**
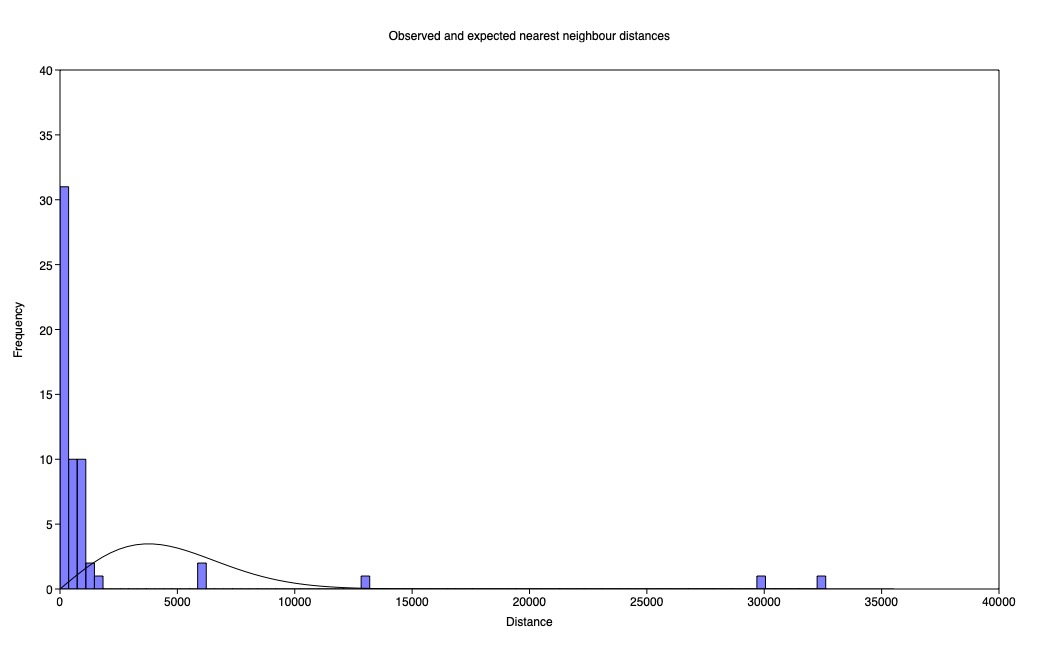
**

Figure 1. Observed and expected nearest neighbor distances between observed presences of *Parochlus steinenii* in the South Shetland Islands, Maritime Antarctica. Analysis and graphics were obtained using Past Software v.3.23 (Hammer, O., Harper, D. & Ryan, P. PAST: Paleontological Statistics software package for education and data analysis. *Palaeontol. Electron.* 4, 9 (2001)).


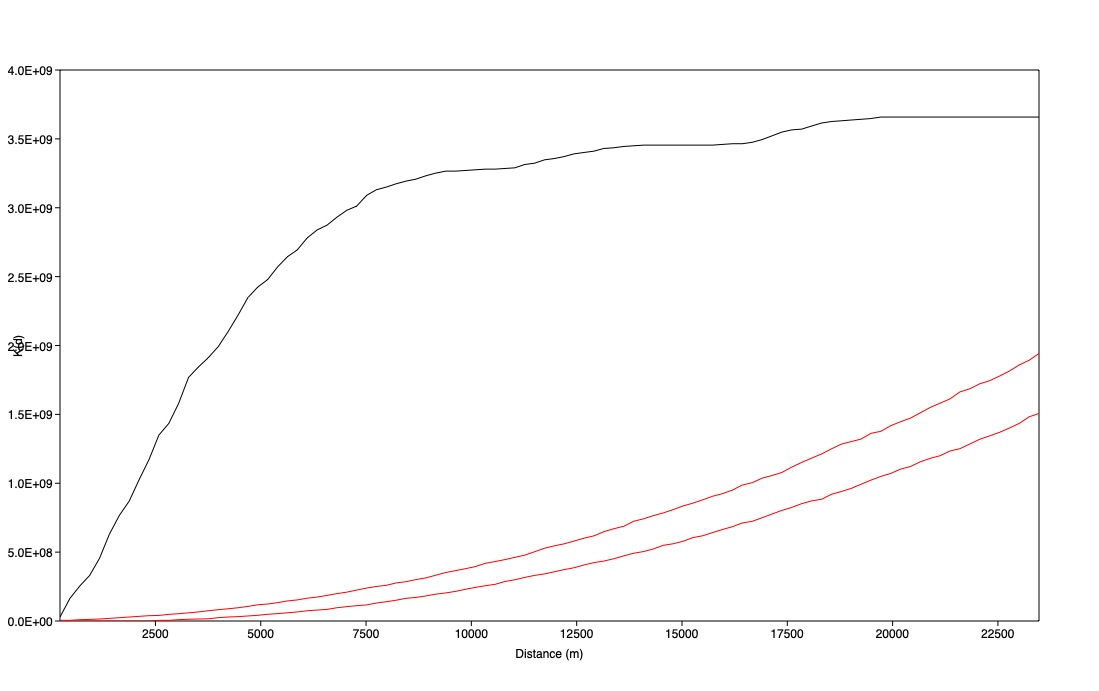


Figure 2. Ripley’s K (d) univariate function for the distribution of *Parochlus steinenii* in the South Shetland Islands. The black line represents the observed distribution, while the red lines in k(d), represent the theoretical distribution under the CSR null hypothesis (montecarlo test, p = 0.0093). Analysis and graphics were obtained using Past Software v.3.23 (Hammer, O., Harper, D. & Ryan, P. Past: Paleontological Statistics Software Package for education and data analysis. *Palaeontol. Electron.* 4, 9 (2001)).

**Welch two sample T-test**

GDLF

Welch Two Sample t-test

data: datos[, 1] and datos[, 2]

t = 32.869, df = 50.008, p-value < 2.2e-16

alternative hypothesis: true difference in means is not equal to 0

95 percent confidence interval:

0.4155884 0.4696861

sample estimates:

mean of x mean of y

0.9964471 0.5538098

ACCESS

Welch Two Sample t-test

data: datos[, 1] and datos[, 2]

t = 38.545, df = 50.013, p-value < 2.2e-16

alternative hypothesis: true difference in means is not equal to 0

95 percent confidence interval:

0.4210735 0.4673697

sample estimates:

mean of x mean of y

0.9972490 0.5530275

BNU

Welch Two Sample t-test

data: datos[, 1] and datos[, 2]

t = 28.367, df = 50.006, p-value < 2.2e-16

alternative hypothesis: true difference in means is not equal to 0

95 percent confidence interval:

0.3859219 0.4447369

sample estimates:

mean of x mean of y

0.9969412 0.5816118

CESM1

Welch Two Sample t-test

data: datos[, 1] and datos[, 2]

t = 37.249, df = 50.007, p-value < 2.2e-16

alternative hypothesis: true difference in means is not equal to 0

95 percent confidence interval:

0.4221139 0.4702312

sample estimates:

mean of x mean of y

0.9966020 0.5504294

CSIRO

Welch Two Sample t-test

data: datos[, 1] and datos[, 2]

t = 32.546, df = 50.007, p-value < 2.2e-16

alternative hypothesis: true difference in means is not equal to 0

95 percent confidence interval:

0.3695299 0.4181407

sample estimates:

mean of x mean of y

0.9974529 0.6036176


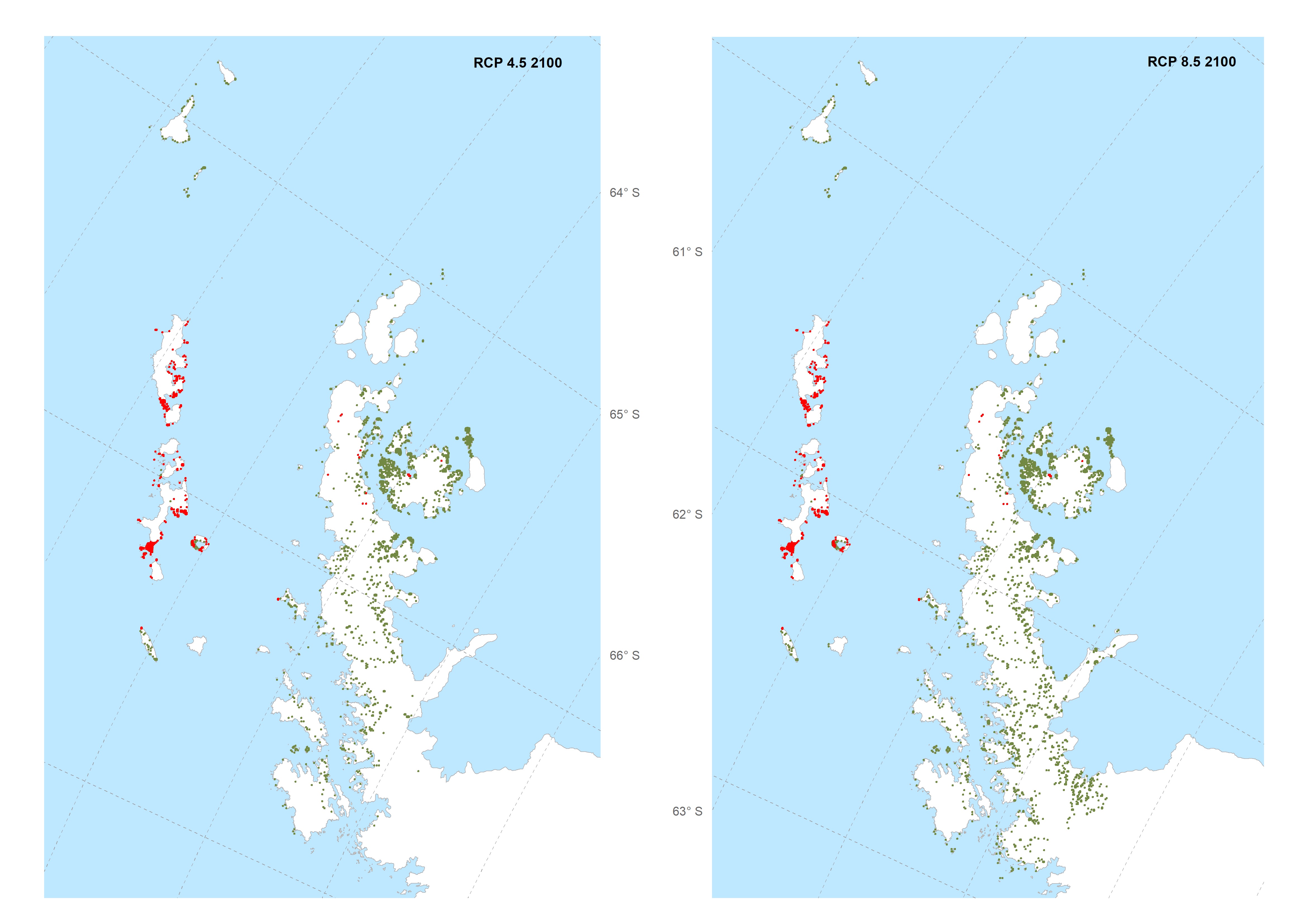


Figure 3. This figure shows appropriate habitats in current time (red dots) and under future scenarios RCP 4.5 and RCP 8.5 for 2100 (green dots). Under both scenarios, *Parochlus steinenii* maintains its present distribution and it could considerably expand its distribution. This map was generated using ArcGIS® software by Esri, ArcMap 10.1. ArcGIS® and ArcMap™ are the intellectual property of Esri and are used herein under license. Copyright © Esri. All rights reserved. For more information about Esri® software, please visit [www.esri.com](http://www.esri.com/).

**
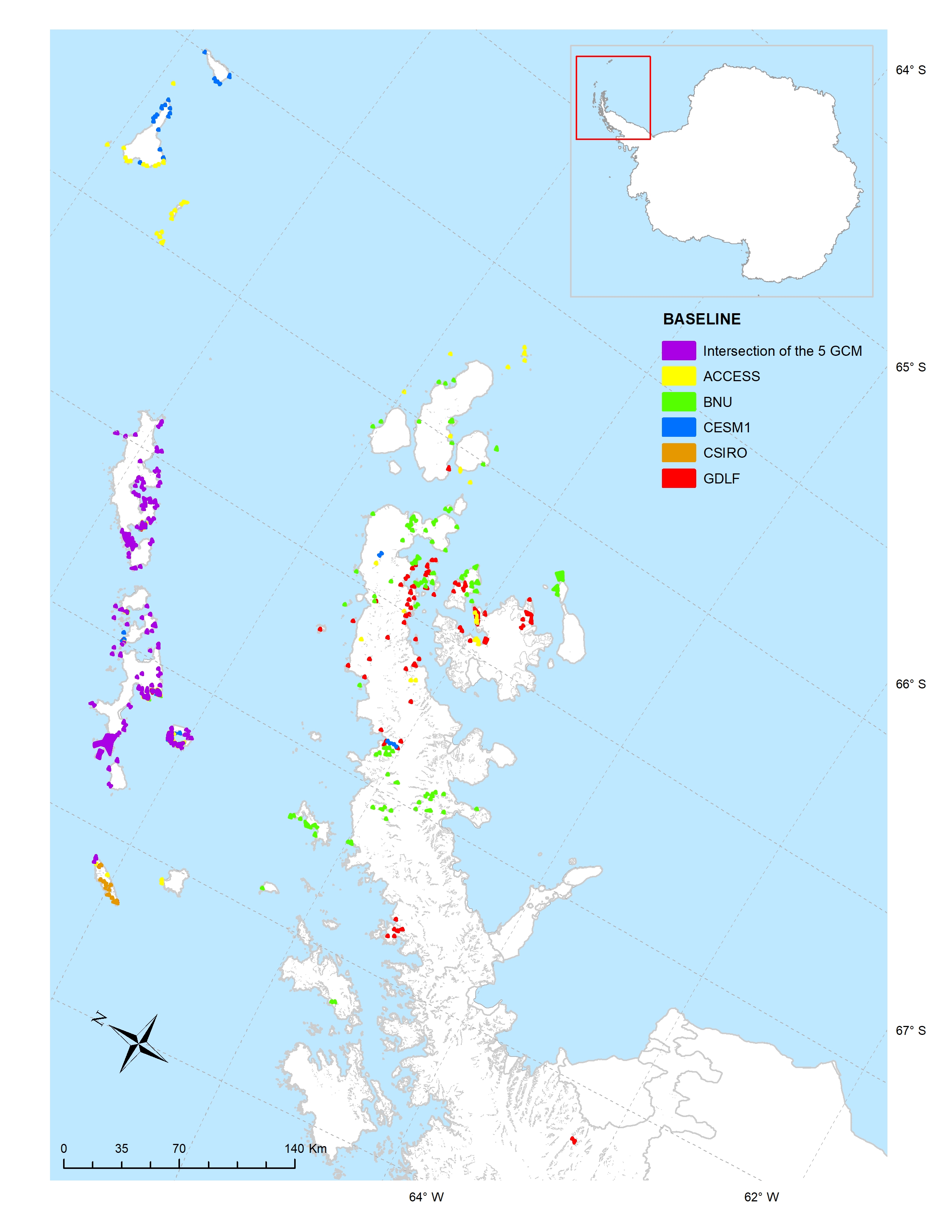
**

Figure 4. Overlapping of the 5 General Circulation Models (GCM). The purple dots indicate the intersection of the 5 models. This intersection is very similar to the baseline distribution obtained from GDLF, the selected model in this study. This map was generated using ArcGIS® software by Esri, ArcMap 10.1. ArcGIS® and ArcMap™ are the intellectual property of Esri and are used herein under license. Copyright © Esri. All rights reserved. For more information about Esri® software, please visit [www.esri.com](http://www.esri.com/).

**
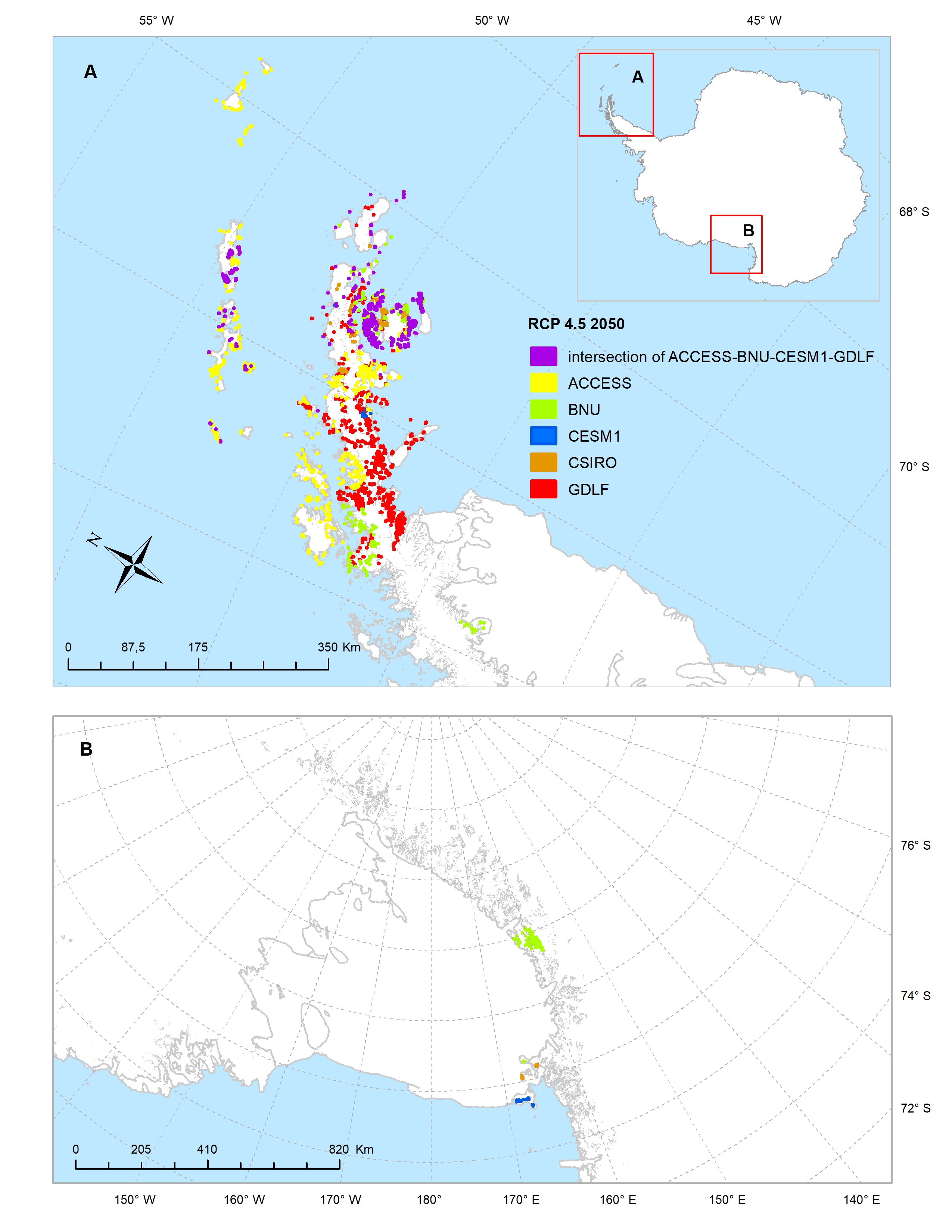
**

Figure 5. Overlapping of the 5 General Circulation Models (GCM) for RCP 4.5 2050. The purple dots indicate the intersection of the 4 models (ACCESS, BNU, CESM1, GDLF). Most models present a distribution that remains between the South Shetland Islands and the Peninsula, nonetheless, BNU, CSIRO, and CESMI show small extension into the continent (Bioregions 9 and 10). This map was generated using ArcGIS® software by Esri, ArcMap 10.1. ArcGIS® and ArcMap™ are the intellectual property of Esri and are used herein under license. Copyright © Esri. All rights reserved. For more information about Esri® software, please visit [www.esri.com](http://www.esri.com/).

**
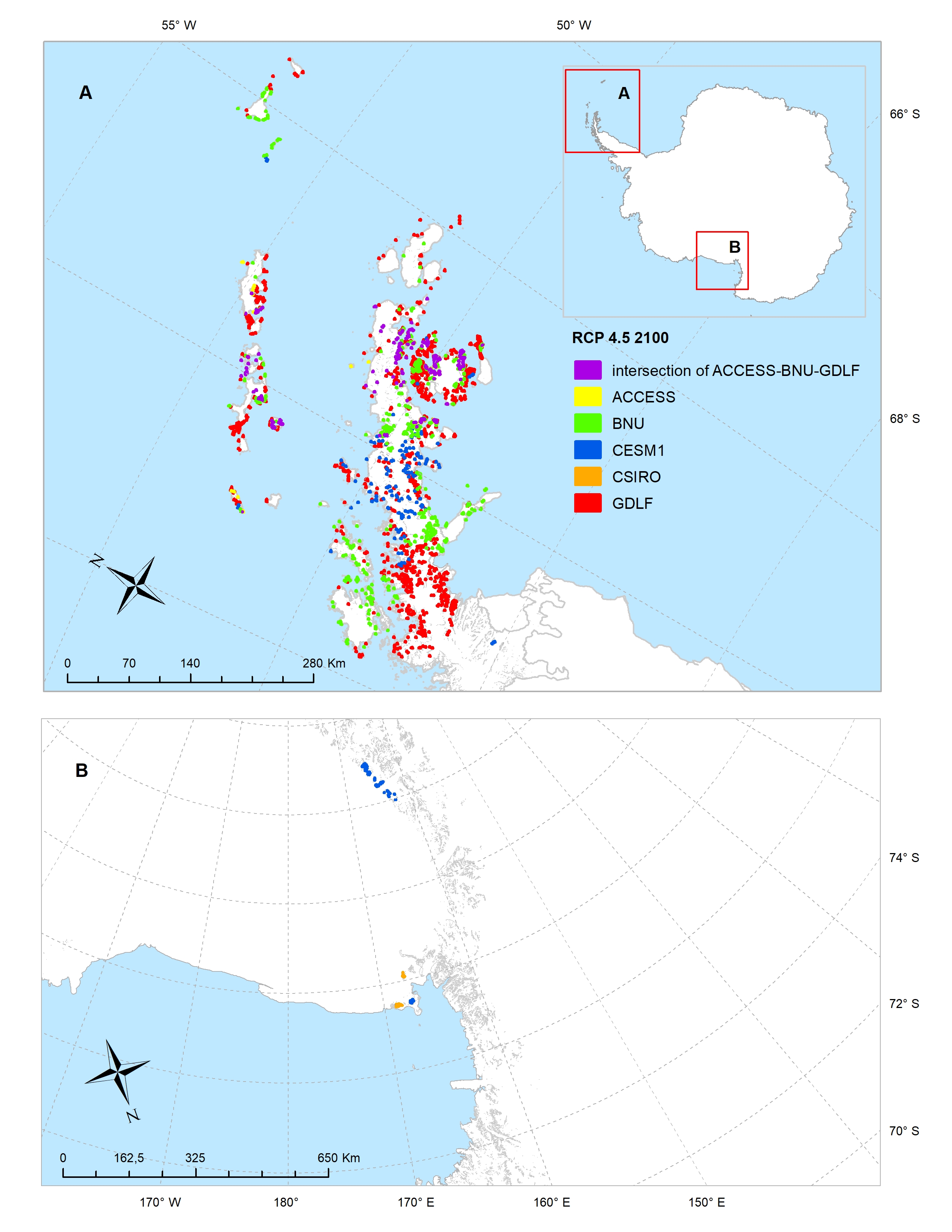
**

Figure 6. Overlapping of the 5 General Circulation Models (GCM) RCP 4.5 2100. The purple dots indicate the intersection of the 3 models (ACCESS-BNU-GDLF). Most models present a distribution that remains between the South Shetland Islands and the Peninsula, nonetheless, BNU, CSIRO, and CESMI show small extension into the continent (Bioregions 9 and 10). This map was generated using ArcGIS® software by Esri, ArcMap 10.1. ArcGIS® and ArcMap™ are the intellectual property of Esri and are used herein under license. Copyright © Esri. All rights reserved. For more information about Esri® software, please visit [www.esri.com](http://www.esri.com/).

**
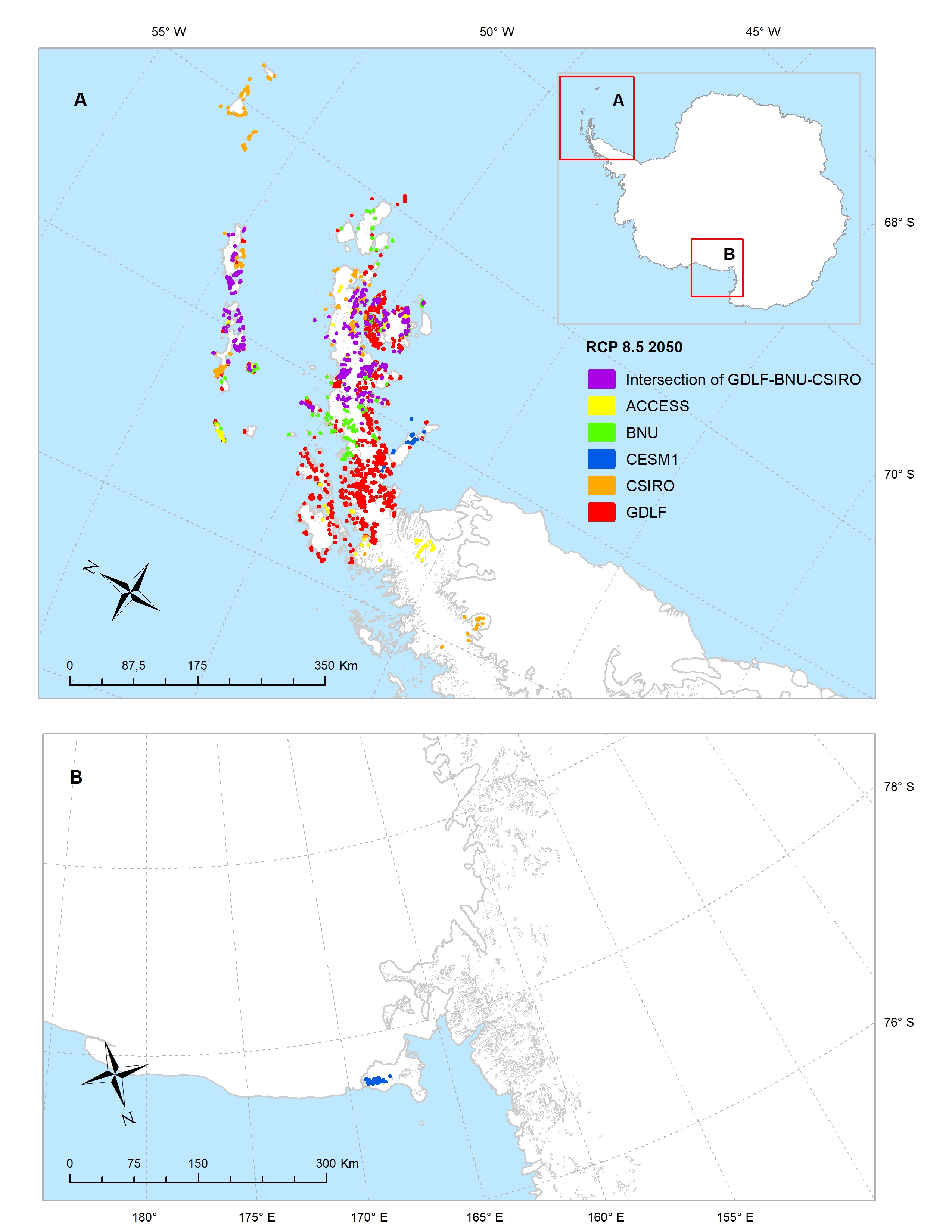
**

## Figure 7. Overlapping of the 5 General Circulation Models (GCM) for RCP 8.5 2050. The purple dots indicate the intersection of the 4 models (GDLF, BNU, CSIRO). Most models present a distribution that remains between the South Shetland Islands and the Peninsula, nonetheless, BNU, CSIRO, and CESMI show small extension into the continent (Bioregion 12). This map was generated using ArcGIS® software by Esri, ArcMap 10.1. ArcGIS® and ArcMap™ are the intellectual property of Esri and are used herein under license. Copyright © Esri. All rights reserved. For more information about Esri® software, please visit [www.esri.com](http://www.esri.com/).

**
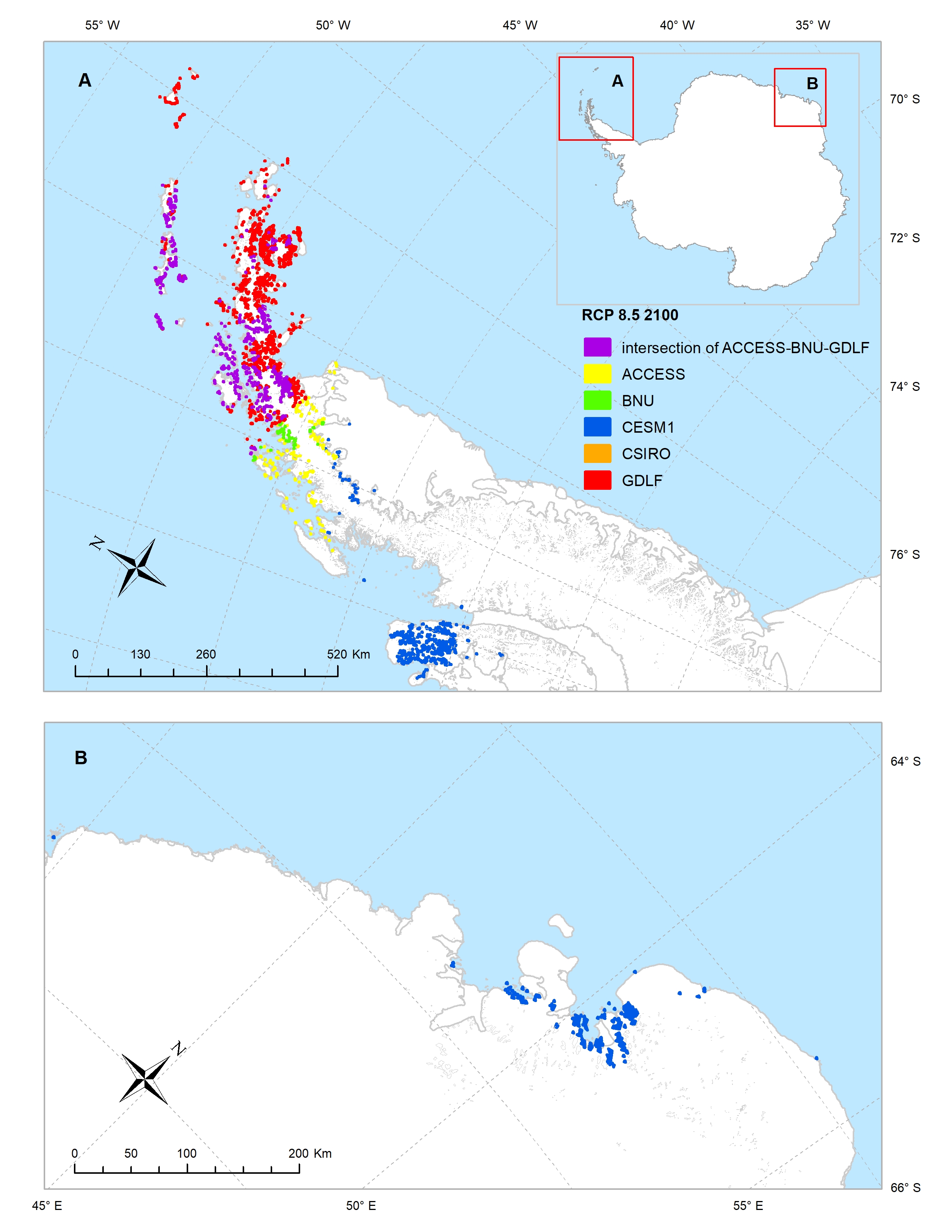
**

## Figure 8. Overlapping of the 5 General Circulation Models (GCM) for RCP 8.5 2100. The purple dots indicate the intersection of the 4 models (GDLF, BNU, CSIRO). Most models present a distribution that remains between the South Shetland Islands and the Peninsula, nonetheless, BNU, CSIRO, and CESMI show small extension into the continent (Bioregion 5). This map was generated using ArcGIS® software by Esri, ArcMap 10.1. ArcGIS® and ArcMap™ are the intellectual property of Esri and are used herein under license. Copyright © Esri. All rights reserved. For more information about Esri® software, please visit [www.esri.com](http://www.esri.com/).

**SD MAPS OF MAXENT FOR THE MODEL SELECTED GDLF**

**
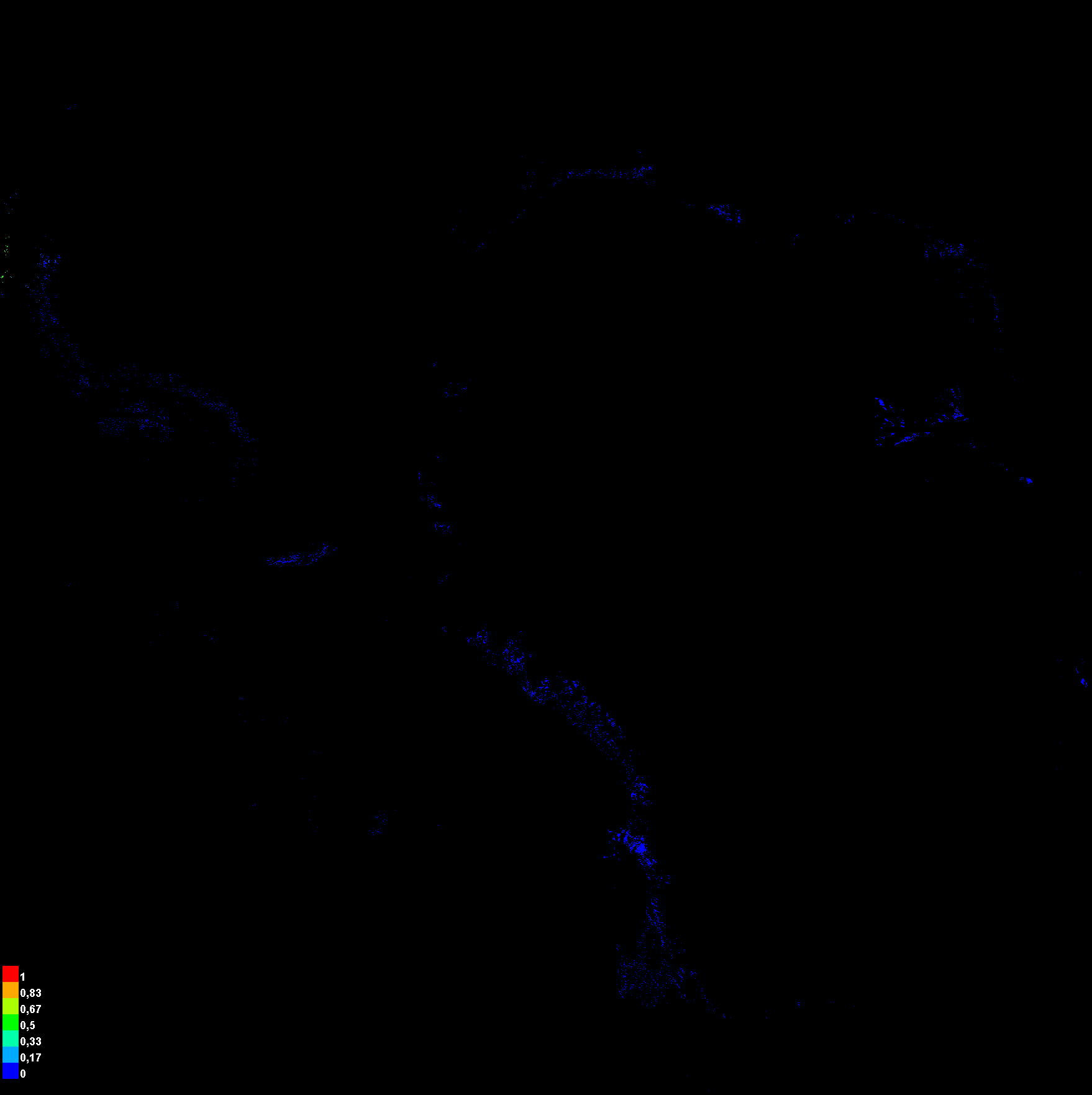
**

Figure 9. Current model for *Parochlus steinenii*

**
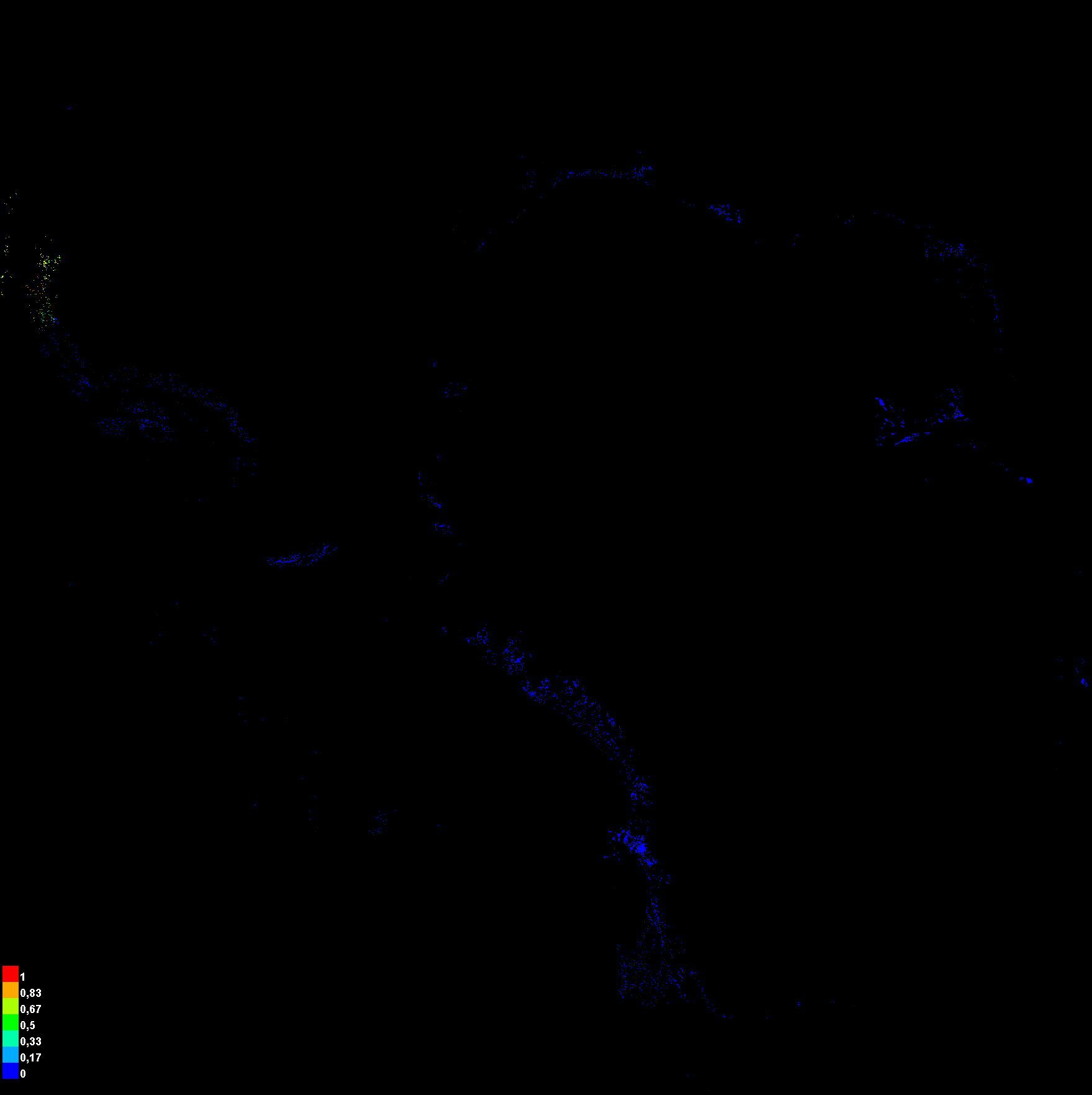
**

Figure10. RCP 4.5 2050 scenario for *Parochlus steinenii*

**
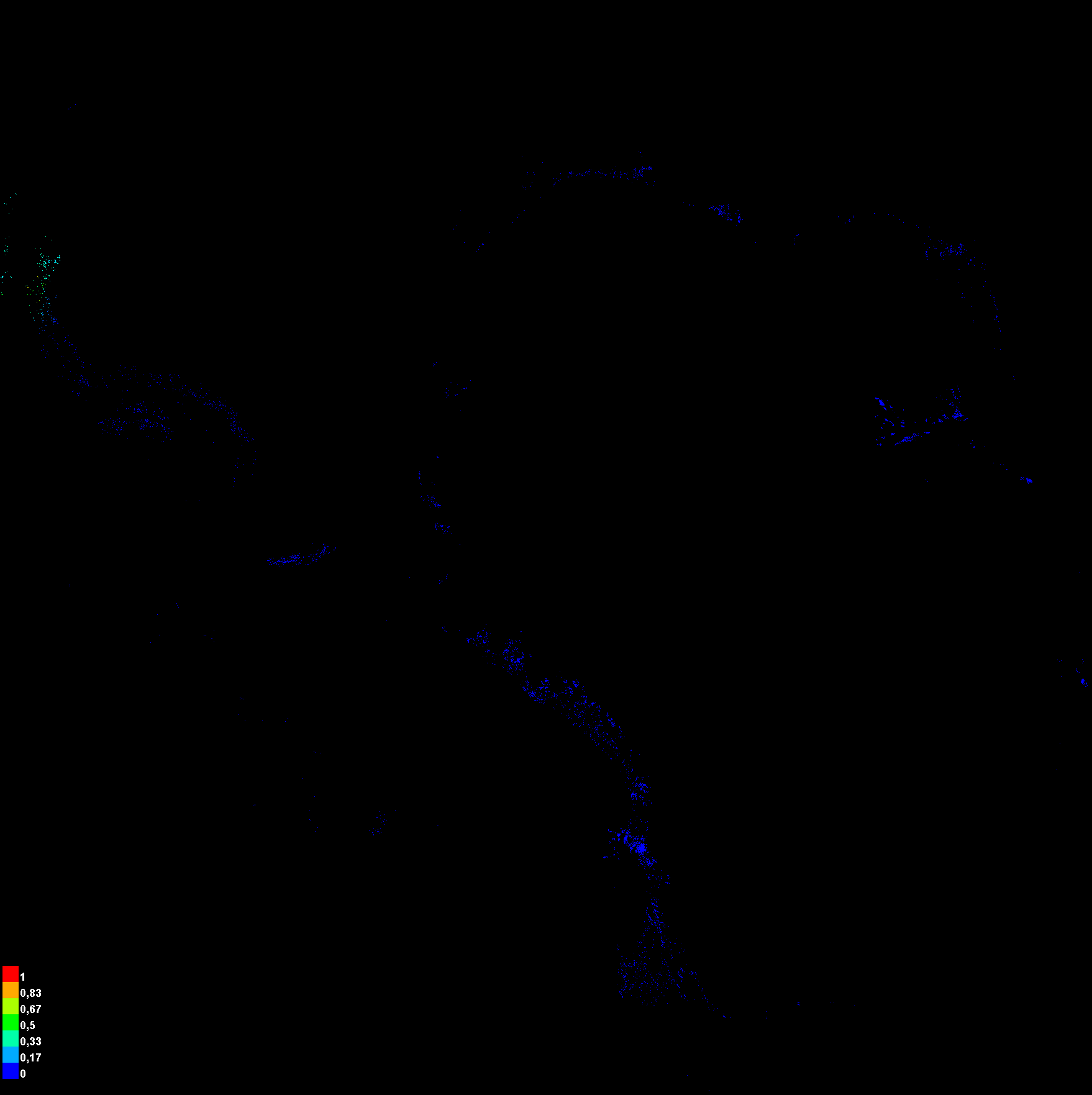
**

Figure 11. RCP 4.5 2100 scenario for *Parochlus steinenii*

**
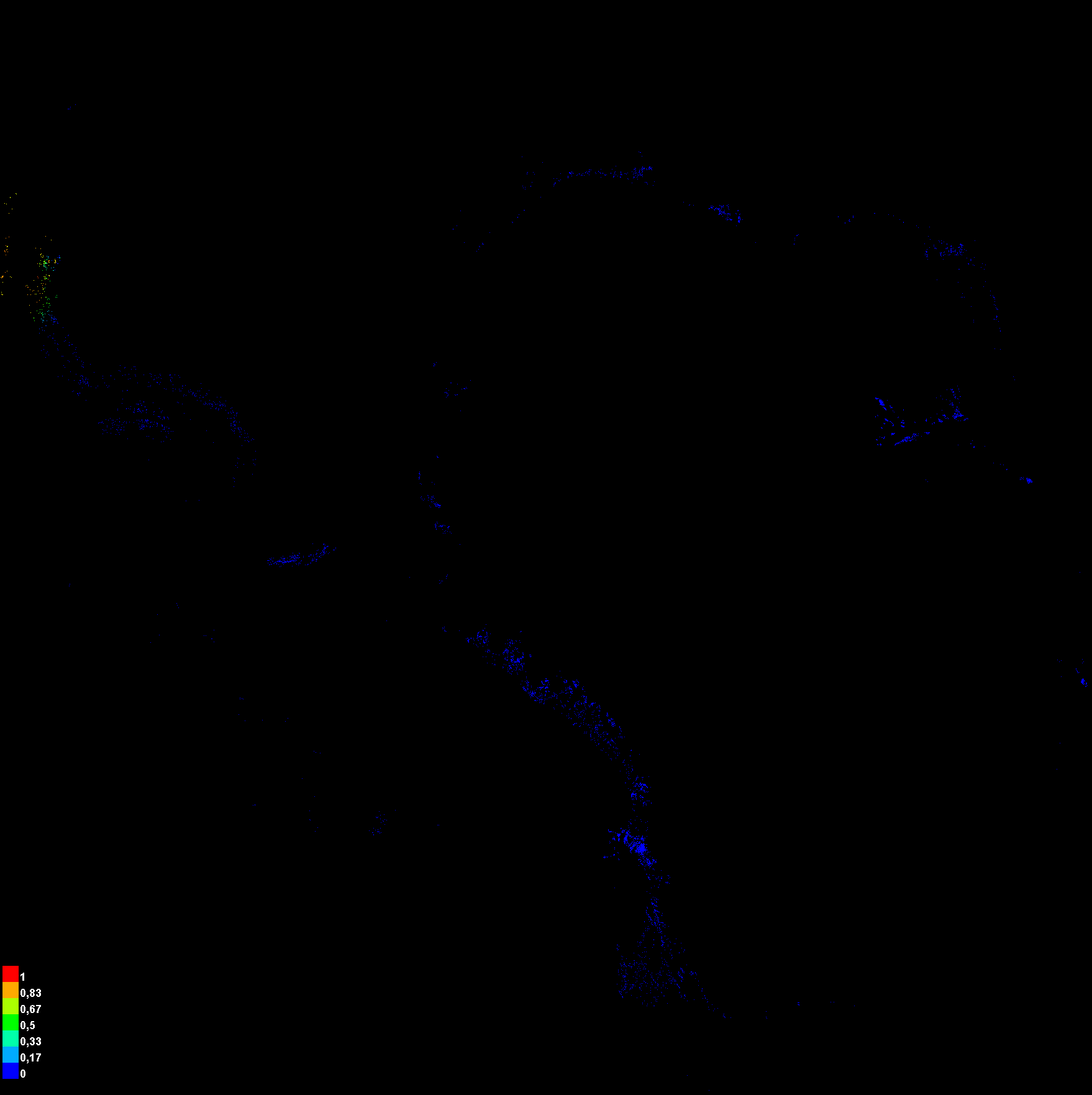
**

Figure 12. RCP 8.5 2050 scenario for *Parochlus steinenii*

**
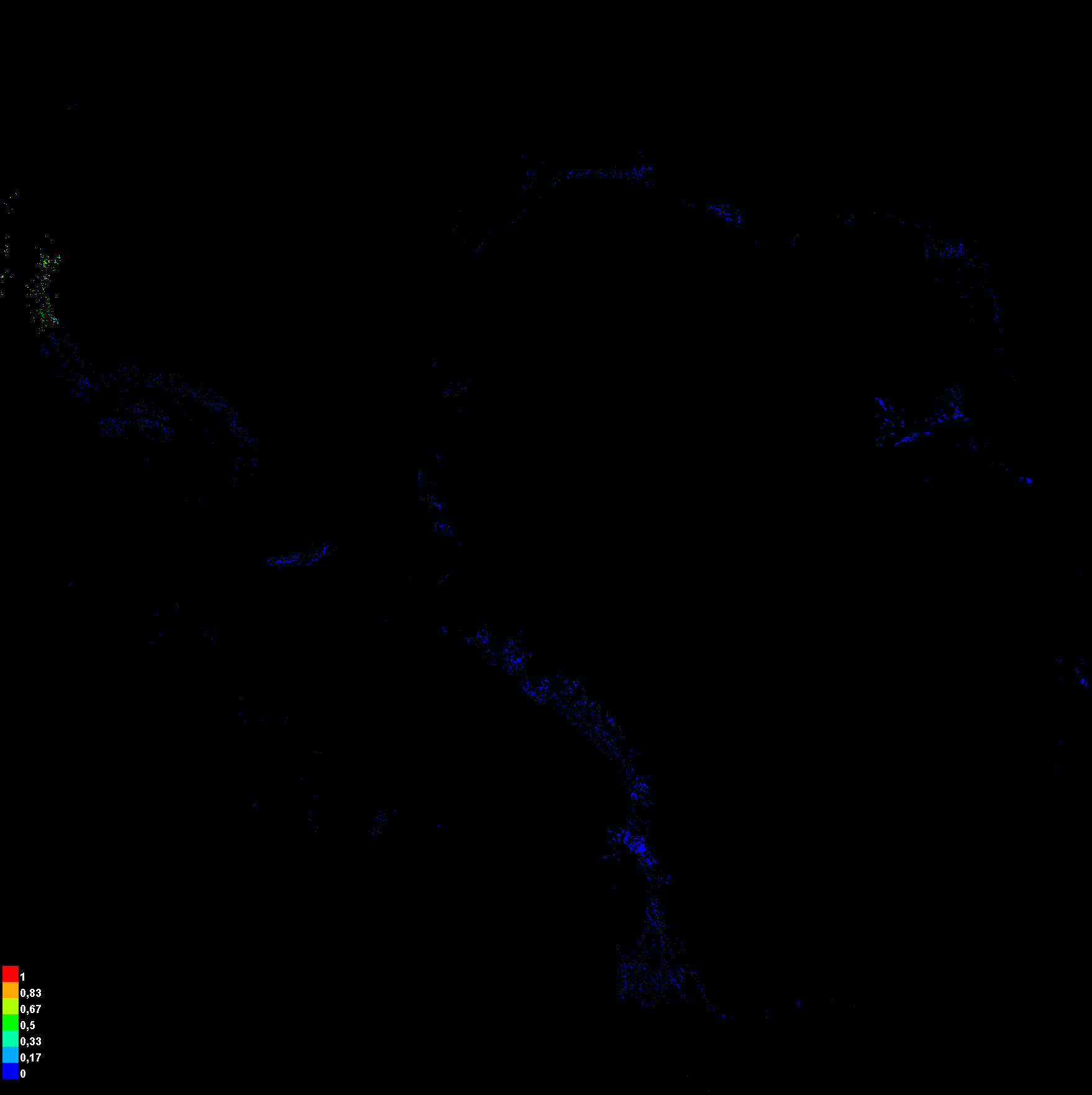
**

Figure 13. RCP 8.5 2100 scenario for *Parochlus steinenii*
